# Identification of *Ziziphus jujuba* cv. Dongzao DNA Demethylase *ZjROS1* Gene Family and Construction of CRISPR/Cas9-Mediated Gene-Editing Vector

**DOI:** 10.3390/genes16020228

**Published:** 2025-02-17

**Authors:** Jiaqi Wang, Huiran Wang, Jiayi Zhai, Fulun Zhu, Yufeng Ren, Jun Zhou, Zhikai Zhang, Lan Luo, Wendi Xu

**Affiliations:** 1 School of Biological Science and Engineering, North Minzu University, Yinchuan 750021, China; wjq01181128@163.com (J.W.); whr947124@163.com (H.W.); 18503790628@163.com (J.Z.); metatron971@outlook.com (F.Z.); ren_yufeng@163.com (Y.R.); zhoujunbo@163.com (J.Z.); zhangzhikai0019@163.com (Z.Z.); luolan020313@163.com (L.L.); 2 Innovation Team for Genetic Improvement of Economic Forest, North Minzu University, Yinchuan 750021, China

**Keywords:** bioinformatics analysis, expression characteristics analysis, gene cloning, *ROS1* gene family, vector construction, *Ziziphus jujuba* cv. Dongzao

## Abstract

DNA methylation is one of the earliest and most extensively studied epigenetic regulatory mechanisms. The *ROS1 (Repressor of Silencing 1)* gene was first discovered in *Arabidopsis thaliana*, and it is a DNA demethylase that can remove 5-methylcytosine from DNA, thereby affecting DNA methylation levels and gene expression. **Objectives**: The objective of this study was to investigate the role of *ROS1* in the development and maturation of *Ziziphus jujuba* cv. “Dongzao” fruit. **Methods**: We cloned the *ROS1* gene and conducted bioinformatics and expression characteristics analyses on it. **Results**: Three *ROS1* genes, named *ZjROS1-1~3*, was identified, and each member protein was localized in the nucleus, cytoskeleton, chloroplast, and vacuole. The promoter contained *cis*-elements such as light response, plant hormone signal transduction, and stress response *cis*-elements, and it interacted with many proteins such as CMT, MET, and ZDP. The results of the real-time fluorescence quantitative PCR show that *ZjROS1* has specific expression patterns in different tissues of *Z. jujuba* cv. Dongzao, and the expression of *ZjROS1-2* in flowers and fruits is high. At the same time, CRISPR/Cas9 technology was used to construct a gene-editing vector for *ZjROS1*, which provided a basis for the subsequent genetic transformation. **Conclusions**: In this study, the biological function of *ZjROS1* was clarified and a gene-editing vector was constructed, which provided a theoretical basis for the regulation mechanism of demethylase *ZjROS1* in the fruit ripening and development of *Z. jujuba* cv. Dongzao.

## 1. Introduction

*Z. jujuba* cv. Dongzao is a cultivar of *Ziziphus* (*Ziziphus jujuba* Mill.) in the Rhamnaceae family and a unique cultivar of late fresh jujuba mill in China [1,2,3]. The flowering period of *Z. jujuba* cv. Dongzao is around June, and the fruit ripening period is usually from late October to early November [2,3,4]. It takes about three and a half months for *Z. jujuba* cv. Dongzao to transition from flowering to fruiting. According to the changes in peel color, this can be divided into three stages: white ripening stage, half-red stage, and full-red stage. When jujube enters the white ripening stage, it begins to mature, and the fruit surface is green, with a low sugar content and high hardness. In the half-red stage, the surface of the jujube fruit begins to color, the flesh is crisp, and the sugar content increases sharply. In the full-red stage, the surface of the jujube fruit is red, the water content of the fruit decreases, and the hardness of the flesh decreases [5].

*Z. jujuba* cv. Dongzao is a type of fresh fruit with important economic value; it is famous for its unique flavor and remarkable nutritional value. In addition to containing various amino acids, vitamins, and minerals, it also contains many functional bioactive ingredients, known as “jujube best”, and this is favored by consumers [6,7,8,9,10]. The formation of the flavor quality of *Z. jujuba* cv. Dongzao fruit is mainly affected by external environmental factors, such as water, light, and temperature. In addition to external environmental factors, fruit quality is also related to the expression of its own regulated genes. Gene expression is subject to genetic and epigenetic regulation. A large number of studies have examined the maturation flavor quality of *Z. jujuba* cv. Dongzao fruit from the aspects of gene regulation, transcriptomics, and metabolomics, such as phosphoenolpyruvate, galactose synthesis pathway genes, and phenolic-synthesis-related regulatory enzymes [11,12,13,14]. Although the mechanisms of DNA methylation and demethylation in *A. thaliana* and some fruits have been elucidated [15,16,17], the regulatory mechanisms of DNA methylation during the development of *Z. jujuba* cv. Dongzao fruit are still poorly understood.

DNA methylation is a common epigenetic pathway in plants and one of the earliest and most extensively studied topics in epigenetics. In plants, the DNA glycosylase *DEMETER* family members *DME*, *DML2*, and *DML3* and silencing suppressor 1 (*ROS1*) perform active DNA demethylation. The *ROS1* gene, first identified in *A. thaliana*, is a DNA demethylase capable of removing 5-methylcytosine from DNA, thereby affecting DNA methylation levels and gene expression. Previous studies have shown that Ros1-mediated DNA demethylation and gene regulation control a variety of processes, including antimicrobial defense, seed dormancy, and stomatal stem cell production. Studies have shown that active DNA demethylation mediated by silencing suppressor 1 (ROS1) in plants plays an important role in regulating the DNA methylation balance in biotic and abiotic stress responses. Research has shown that DNA demethylase ROS1 can negatively regulate the RdMM pathway [18,19]. The DNA demethylation of ROS1 in the RdMM targeting region plays an important role in regulating gene expression [20]. ROS1-mediated DNA demethylation in *Arabidopsis* antagonizes the RddM pathway to balance DNA methylation levels and regulate gene expression [21].

In recent years, new technologies, as represented by the CRISPR/Cas9 system, have rapidly expanded the research and application fields of gene editing [22]. The CRISPR/Cas9 system is an immune mechanism widely present in bacteria and archaea [23]. To date, CRISPR/Cas9 gene-editing and related derivative technologies have been widely used in the genetic improvement of crops. At present, it is mainly used to improve crop yield, quality, and disease resistance. Studies have shown that knocking out the circular E3 ligase-encoding gene *TaGW2* can increase the wheat grain length and width, thereby increasing the wheat yield [24,25]. By knocking out the *Wx1* gene through the CRISPR/Cas9 system, the amylopectin content in maize can reach close to 100% [26,27], and other phenotypes do not change. The knockout mutation obtained from the CRISPR/Cas9-targeted editing of the *SlMlo1* gene in tomato can produce resistance to powdery mildew [28]. Feng Zhijuan et al. took the conserved domain of the *GmGBSSⅡ* protein as the target region to construct a CRISPR/Cas9 gene-editing vector for the *GmGBSSⅡ* gene in vegetable soybean and provided experimental materials for genetic transformation to obtain *GmGBSSⅡ* gene-edited mutants in vegetable soybean [29]. At present, there are relatively few studies on CRISPR/Cas9-mediated gene editing in jujube. However, with the gradual improvement in the genetic transformation system of jujube and the development of related technologies, its application prospects are broad. Some progress has been made in research on the *ROS1* gene using CRISPR/Cas9-mediated gene editing. Researchers carried out CRISPR/Cas9-mediated gene editing on the *OsROS1* gene in rice. They designed two 20 bp guide RNAs targeting the first and fifteenth exons of the *OsROS1* gene, constructed knockout vectors, and introduced them into rice varieties through *Agrobacterium*-mediated transformation. As a result, multiple *OsROS1* knockout mutants were obtained. Most of the mutants showed sterility during the reproductive growth stage, and the seed-setting rates of the few mutants that could produce seeds were also significantly reduced. This indicates that the *OsROS1* gene is crucial for the development of rice pollen and embryo sacs [30]. To explore the role of the *CmROS1* gene in fruit ripening and epigenetic regulation in melon, researchers used CRISPR/Cas9 technology to generate homozygous knockout mutants of *CmROS1* under the climacteric genetic background of melon. The results show that, compared with the wild type, the mutants displayed premature ethylene production and altered climacteric behavior, indicating that the *CmROS1* gene plays a role in the climacteric process of melon fruits. In addition, an analysis of the single-cytosine methylome of the *CmROS1* knockout mutants revealed DNA methylation changes in the promoter regions of key ripening genes (such as *ACS1*, *ETR1*, and *ACO1*) and ripening-related transcription factors (such as *NAC-NOR*, *RIN*, and *CNR*). This indicates that *CmROS1*-mediated DNA demethylation is of great significance for triggering the ripening of melon fruits [31].

With the rapid development of high-throughput sequencing technology and the continuous progress of genome-wide DNA methylation research methods, an increasing number of studies have been conducted on the changes in and mechanisms of DNA methylation during fruit ripening [32]. DNA methylation has been extensively studied with regard to fruit quality because it is involved in anthocyanin accumulation [33], fruit flavor formation, and fruit development and ripening [15,16,34]. In *A. thaliana*, previous studies have found that the *ROS1* gene has a significant impact on plant growth and development. In *A. thaliana*, *ROS1* plays a crucial regulatory role in the process of responding to abiotic stress. It promotes the expression of defense genes and stress-responsive genes by reducing the DNA methylation level. These genes are involved in physiological processes such as plant antioxidant defense, osmotic regulation, and ion balance, helping plants better cope with abiotic stress [35]. In *Malus pumila*, researchers used RNA-sequencing technology to detect the transcriptional levels of the *MdROS1* gene related to anthocyanin content and genes related to anthocyanin biosynthesis in apples under low-temperature stress conditions. The results showed that transient silencing of *MdROS1* in apple leaves and fruits could inhibit anthocyanin accumulation and lead to a decrease in the expression of anthocyanin biosynthesis genes, while the opposite was true for leaves and fruits overexpressing *MdROS1*. This indicates that *ROS1* affects the anthocyanin biosynthesis pathway and increases anthocyanin accumulation by reducing the methylation level of the promoters of anthocyanin-related genes, thus influencing aspects such as plant color development [36]. Taking the model organism *A. thaliana* as the research object, Academician Zhu Jiankang’s research team revealed through whole-genome bisulfite sequencing (WGBS) and other analyses that mutations in the DNA demethylase *ROS1* led to a generation-by-generation increase in the DNA methylation level at the genomic level. This study demonstrated that *ROS1* plays an important monitoring function in preventing the trans-generational inheritance of randomly formed DNA methylation and maintaining the stability of the epigenome. Since the stability of the epigenome is crucial for the normal development of plants, it indirectly illustrates that the *ROS1* gene has a significant impact on plant development [37].

In this experiment, the DNA methylation sites of the *Z. jujuba* cv. Dongzao genome were cloned and sequenced, and *ROS1*-related genes were successfully isolated. Although the formation mechanism in *Z. jujuba* cv. Dongzao fruit ripening has been studied, many questions remain. For example, how do *ROS1*-related genes regulate DNA methylation levels to participate in regulating the expression levels of genes involved in fruit development, thereby affecting fruit ripening? What are the expression characteristics of the *ROS1* gene in *Z. jujuba* cv. Dongzao? Therefore, in this study, the function of *ZjROS1* in the development of *Z. jujuba* cv. Dongzao fruit was studied by cloning and identifying the *ZjROS1* gene family and conducting serial analyses. Meanwhile, a *ZjROS1* gene-editing vector for *Z. jujuba* cv. Dongzao was constructed using CRISPR/Cas9 technology. This study lays a foundation for further studies on the function of *ZjROS1* in *Z. jujuba* cv. Dongzao and the establishment of a *ZjROS1* genetic transformation system.

## 2. Materials and Methods

### 2.1. Materials

The plant materials used in this study were obtained from 3-year-old *Z. jujuba* cv. Dongzao, and wild *Ziziphus jujuba* was used as the grafting rootstock. These plants were planted at the Institute of North Minzu University (latitude 38°29′ N, longitude 106°10′ E), with a light cycle of 12 h/d and a light intensity of 150 μmol/(m^2^·s). The young leaves, old leaves, young stems, old stems, flowers, and fruits of the plants at three different stages, namely, the white ripening stage, semi-red stage, and full-red stage, were collected in different seasons. After picking, they were quickly frozen in liquid nitrogen and then taken back to the laboratory for storage in a −80 °C refrigerator [38].

### 2.2. Research Methods

#### 2.2.1. Bioinformatics Analysis of the *ZjROS1* Gene Family

##### Gene Family Identification and Protein Physicochemical Property Analysis

The *AtROS1* gene sequences were obtained from the *A. thaliana* TAIR database (https://www.arabidopsis.org/ (accessed on 27 January 2024)). The whole-genome and protein sequences of jujube were obtained from NCBI. TBtools was used to perform local BLAST and extract the sequences to obtain the genes related to demethylase *ROS1* in *Z. jujuba* cv. Dongzao. SMART was used to identify the ROS1 protein sequence in order to ensure that all genes contained the ROS1 domain. The protein sequences were input into the Protparam webpage (https://web.expasy.org/protparam/ (accessed on 28 January 2024)) of the Expasy website (https://www.expasy.org/ (accessed on 28 January 2024)). The molecular weight, isoelectric point, amino acid composition, hydrophobicity, and other related physical and chemical properties of the *ZjROS1* gene family members were analyzed, and the results were calculated and plotted.

On the ProtScale online website (https://web.expasy.org/protscale/ (accessed on 1 February 2024)), “protein sequence” was input, and then “Hydropath./Kyte & Doolittle” was selected for submission to conduct hydrophilicity and hydrophobicity analyses. By inputting the protein sequence into the NetPhos 3.1 Server website (https://services.healthtech.dtu.dk/services/NetPhos-3.1/ (accessed on 1 February 2024)) and submitting it, the analysis and prediction results of the protein phosphorylation sites were obtained.

##### Prediction of Signal Peptides and Transmembrane Domains, Chromosome Localization, and Subcellular Localization

The protein sequences were input into the SingalP-3.0 Server website (https://services.healthtech.dtu.dk/services/SignalP-3.0/ (accessed on 2 February 2024)), and the signal peptide prediction results of the protein sequence were obtained. The protein sequences were input into the TMHMM Server v.2.0 website (http://www.cbs.dtu.dk/services/TMHMM/ (accessed on 2 February 2024)), and the prediction results from the analysis of the proteins’ transmembrane structure field were obtained.

The gff annotation file for jujube was downloaded from NCBI. The chromosome length and location information of each gene were searched for and determined. A chromosome location map of the *ZjROS1* gene family was drawn using MG2C.V2.2 (http://mg2c.iask.in/mg2c_v2.1/ (accessed on 2 February 2024)). The protein sequence was pasted on the WoLF PSORT website (https://wolfpsort.hgc.jp/ (accessed on 2 February 2024)). After choosing “plants” and submitting, the prediction results of the subcellular localization of the protein sequence were obtained.

##### Analysis of Conserved Motif and Conserved Domain of *ZjROS1* Gene and Prediction of the Secondary and Tertiary Structures of the ZjROS1 Protein

The annotation information of the jujube genome structure was downloaded from NCBI, and MEME (http://meme-suite.org/tools/meme (accessed on 3 February 2024)) was used to predict the conserved structural elements of the ZjROS1 protein sequence. The parameters were set to default. Batch-CDD in NCBI was used to predict the conserved domain of the ZjROS1 protein sequence and was visualized using TBtools. The online website NPS@ (https://npsa-prabi.ibcp.fr/cgi-bin/npsa_automat.pl?page=/NPSA/npsa_hnn.html (accessed on 3 February 2024)) was used to predict the secondary structure of the ROS1 protein sequence. The online website SWISS MODEL (https://robetta.bakerlab.org/ (accessed on 3 February 2024)) was used to predict the tertiary structure of the ROS1 protein sequences.

##### Promoter *Cis*-Acting Element Analysis and Protein Interaction Prediction Analysis

TBtools was used to extract nucleotide data from the first 2000 bp of the promoter region of the *ZjROS1* gene family. The website PlantCARE (http://bioinformatics.psb.ugent.be/ (accessed on 3 February 2024)) was used to predict the *cis*-elements in the *ZjROS1* promoter regions, and the visualization analysis results obtained using TBtools were organized and input.

The genomes of *A. thaliana*, *Nicotiana tabacum*, *Glycine soja*, *Asparagus officinalis*, *Prunus salicina*, *Manihot esculenta*, *Camelina sativa*, and *Helianthus annuus* were used to search for genes of the same family through BLAST. MEGA 7 v7.0..26 software was used to perform multiple sequence alignments on their ROS1 protein sequences and three *Z. jujuba* cv. Dongzao ROS1 protein sequences, and a phylogenetic tree was constructed using the maximum likelihood method. The STRING online website (https://cn.string-db.org/ (accessed on 3 February 2024)) was used for a protein interaction prediction analysis, and the model plant *A. thaliana* was selected as a parameter.

#### 2.2.2. Cloning of *ZjROS1* Gene

The total RNA was extracted from different parts and at different stages of the *Z. jujuba* cv. Dongzao fruits using a plant total RNA extraction kit provided by Beijing ComWin Biotech Co., Ltd. (Beijing, China). The RNA concentration was detected using a microspectrophotometer, and the extraction quality was determined using 1.5% agar gel electrophoresis. A PrimeScript™ RT reagent kit with a gDNA Eraser (Perfect Real Time) kit (TaKaRa BIO INC, Beijing, China) was used to reverse transcribe the total RNA into cDNA, which was stored at −80 °C for later use.

The CDS sequence of *Z. jujuba* cv. Dongzao was obtained from NCBI, and the *ZjROS1* clone and quantitative primer were designed using the software Primer Premier 5.0. *ZjACT* was selected as the internal reference gene, and the primer sequences were designed and sent to Sangon Biotech (Shanghai) Co., Ltd. (Shanghai, China) for synthesis (Table 1). The cDNA of various parts of *Z. jujuba* cv. Dongzao was amplified using PCR (Supplementary Table S1), the target bands were recovered using 1% agarose gel electrophoresis, and positive clones were identified and sent for testing.

#### 2.2.3. Tissue-Specific Gene Expression Characteristics of *ZjROS1* Family

The internal reference gene *ZjACT* and 3 *ZjROS1* genes were selected, and the young leaves, old leaves, young stems, old stems, flowers, and fruits at the white maturity stage, half-red stage, and full-red stage were selected as cDNA templates. TB Green Premix Ex Taq II (Tli RNaseH Plus) (TaKaRa BIO INC, Beijing, China) was used for real-time fluorescence quantitative PCR (Supplementary Table S2). The experiment was repeated three times for each *ZjROS1* gene. The relative expression levels of the *ZjROS1* gene in various parts and periods of the fruit were calculated using the −ΔΔCT method, and an electronic expression heat map was drawn.

#### 2.2.4. Construction of *Ziziphus jujuba* cv. Dongzao *ZjROS1* Gene-Editing Vector

The online tool CRISPR2 (http://cbi.hzau.edu.cn/cgi-bin/CRISPR2/SCORE (accessed on 5 April 2024)) was used to design a pair of 20 bp left–right oligo DNA strands in the target DNA area. Two primers with high scores were selected and designed in accordance with the design requirements of target site primers for the pP1C.4 carrier (dicotyledon) of Genloci Biotechnologies Inc., Nanjing, China. Thereafter, the primers were sent to Sangon Biotech (Shanghai) Co., Ltd., for synthesis. The purification level was PAGE (Table 2).

Using the carrier as the template, all of the above primers were used with 2×TransStart KD Plus PCR SuperMix (Beijing TransGen Biotech Co., Ltd., Beijing, China), a high-fidelity PCR amplification enzyme, to obtain the sgRNA box (Supplementary Table S3). The PCR products were detected using 1% agarose gel electrophoresis. The target fragments were recovered, and the OD_260_/OD_280_ values of the recovered products were between 1.8 and 2.0. The pP1C.4 vector was digested with EcoRI and XbaI double enzymes (Supplementary Table S4). DNA recombinase was used to recombine the digested product and the amplified fragment recovered above, constructing the recombinant vector (Supplementary Table S5).

The recombinant products were transformed into *Escherichia coli* GT115 (Beyotime Biological Co., Ltd., Shanghai, China) competent cells. The suspended bacterial solution was spread on LB medium containing kanamycin and cultured overnight at 37 °C. Single colonies were selected and verified using PCR. After verification, the culture was expanded, and the bacteria were preserved with 50% glycerol. The positive bacteria were extracted and transferred into *Agrobacterium* GV3101 competent cells (Beyotime Biological Co., Ltd., Shanghai, China). The cells were spread on Kanamycin–Rifampicin Selective (Beyotime Biological Co., Ltd., Shanghai, China) LB solid medium and cultured at 28 °C for 36 h. Single colonies were selected for PCR detection. The selected positive single colonies were placed in kanamycin–rifampicin-resistant LB liquid medium, cultured at 28 °C for 36 h, protected with 70% glycerol, and stored in a −80 °C refrigerator for subsequent genetic transformation.

## 3. Results

### 3.1. Bioinformatics Analysis of the ZjROS1 Gene Family

#### 3.1.1. Gene Family Identification and Protein Physicochemical Property Analysis

By comparing the *Z. jujuba* cv. Dongzao genome with *AtROS1*, using the homologous sequence principle, the genes related to demethylase *ROS1* in *Z. jujuba* cv. Dongzao were searched. Finally, three *ZjROS1* gene sequences, named *ZjROS1-1*, *ZjROS1-2*, and *ZjROS1-3*, were obtained. The physical and chemical properties are shown in Table 3. The numbers of amino acids were 1758, 1938, and 753. The isoelectric points were between 5.0 and 8.0. The instability coefficient was greater than 40, indicating that all were unstable proteins. The fat solubility coefficients were all less than 80, and the total average hydrophilicity was less than 0, indicating that all the proteins were hydrophilic proteins (Table 3 and Supplementary Figure S1). According to the prediction analysis of those protein phosphorylation sites (Supplementary Figure S2), the family proteins all contained phosphoric acid sites in serine, threonine, and tyrosine, among which the amino acids above 0.5 were phosphorylated amino acids based on the pink line (0.5) in the horizontal coordinate. It can be seen that the proteins of the gene family have multiple phosphorylation sites and may be regulated by phosphorylation.

#### 3.1.2. Transmembrane Domain Analysis and Chromosome Localization Prediction

**Figure 1:**
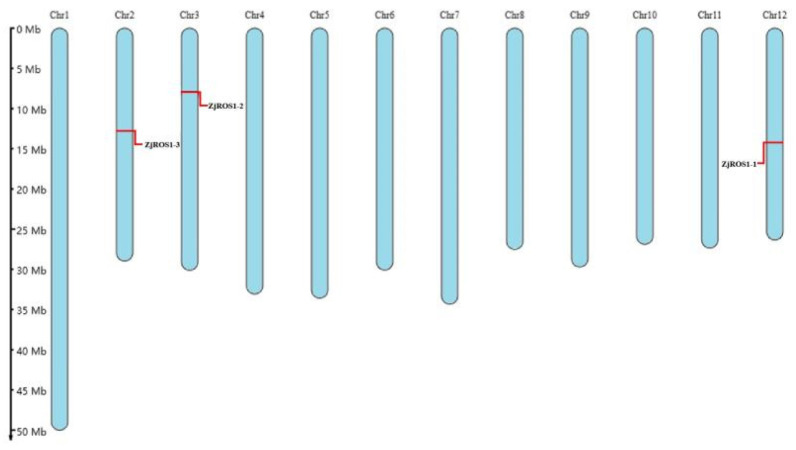
Chromosomal mapping of the *ZjROS1* gene family.

The *ZjROS1* family proteins had no signal peptide (Supplementary Figure S3). The N-best prediction results in the 1-1.2 range showed that the *ZjROS1* family proteins had no transmembrane domain (Supplementary Figure S4), indicating that they were not membrane proteins or secreted proteins. The three genes of *ZjROS1* were distributed on chromosomes 12, 3, and 2, suggesting that the gene distribution of this family is unbiased and that there is no tandem replication (Figure 1) [39]. According to a subcellular localization prediction analysis, *ZjROS1-1* was located in the nucleus; *ZjROS1-2* was located in the nucleus and cytoskeleton; and *ZjROS1-3* was located in the nucleus, cytoskeleton, vacuoles, and chloroplasts (Supplementary Table S6).

#### 3.1.3. *ZjROS1* Gene Conserved Motif, Conserved Domain Analysis, and Secondary and Tertiary Structures’ Prediction Analysis

**Figure 2:**
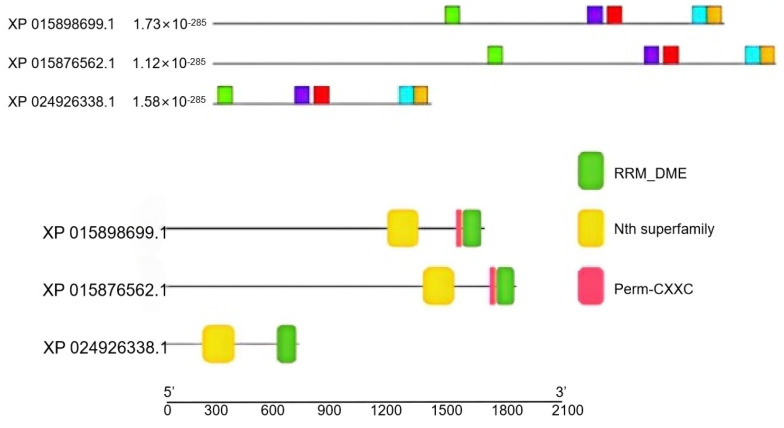
Schematic diagram of the conserved motifs and conserved domain of the ZjROS1 family. (The upper part of the picture: small boxes of different colors or shapes represent different types of conserved motifs.).

According to Figure 2, it was predicted that the arrangement orders of the conserved motifs in the different members of the *ZjROS1* gene family are basically the same. Using Batch-CDD in NCBI to predict conserved domains, it was found that the *RRM_DME* and *Perm-CXXC* complex domains exist in the *ZjROS1* gene family. *RRM_DME* is a predictive *RRM* folding domain involved in DNA demethylation in plants. *Perm-CXXC* is a single unit of the *ZF-CXXC* domain detected in plant proteins and *ROS1* that catalyzes the release of 5-methylcytosine from DNA through the glycosylase/lyase mechanism.

According to the analysis, there were the following four types of secondary structural elements, with random curling and the α-helix accounting for the largest proportions: random curling accounted for 41.97~45.34%; the α-helix accounted for 32.31~35.99%; the extended chain accounted for 14.04~15.47%; and the β-fold accounted for the smallest proportion, ranging from 6.66% to 7.97% (Table 4 and Supplementary Figures S5 and S6).

#### 3.1.4. Promoter *Cis*-Acting Element Analysis and Protein Interaction Prediction Analysis

**Figure 3:**
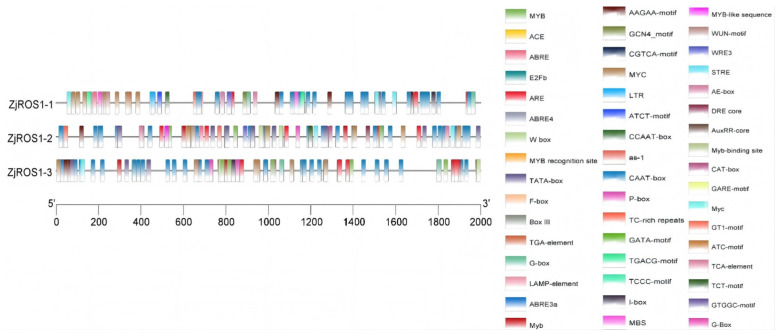
Homeopathic element distribution in the promoter region of the *ZjROS1* gene. The colored boxes indicate the *cis*-element types. Different colors represent different numbers of *cis*-elements.

According to the analysis results in Figure 3, the promoters of the *ZjROS1* gene family members contain various *cis*-acting elements. These include the CAAT-box, TATA-box, and CCAAT-box, which are essential for the transcription initiation sites of promoters. The components involved in the light response are the AAAC-motif, GT1-motif, ACE, G-Box, ATCT-motif, Ctt-motif, I-box, GATA-motif, CTC-motif, AE-box, ATC-motif, GTGGC-motif, and ATCT-motif. The transcription activator is E2Fb. The MYB binding sites are involved in the drought-related element MBS. The antioxidant reaction element is ARE. The cold-stress response element is the W-box. MYB, MYB-like sequences, and MYC elements respond to stress and hormone stimuli. The hormone-related regulatory elements include the plant hormone signal transduction element F-box, AuxRR-core, and TGA-element. The response elements for methyl jasmonate are the CGTCA-motif and TGACG-motif. The salicylic acid response element is the TCA-element. The ABRE, AAGAA-motif, and LAMP-element are also present. The gibberellin response elements are the GARE-motif and P-box. TC-rich repeats, which are defensive stress elements, are related to biotic and abiotic stresses. The element regulating anaerobic induction is ARE. The GCN4-motif *cis*-element is involved in endosperm-specific expression. The WUN-motif is an active element involved in the wound response. The element participating in the stress response is STRE. The dehydration reaction element is the DRE-core. The element participating in the low-temperature response is LTR. The meristem-specific element is the CAT-box. Additionally, WRE3 is the TCF protein-binding site.

#### 3.1.5. Construction of Phylogenetic Tree

**Figure 4:**
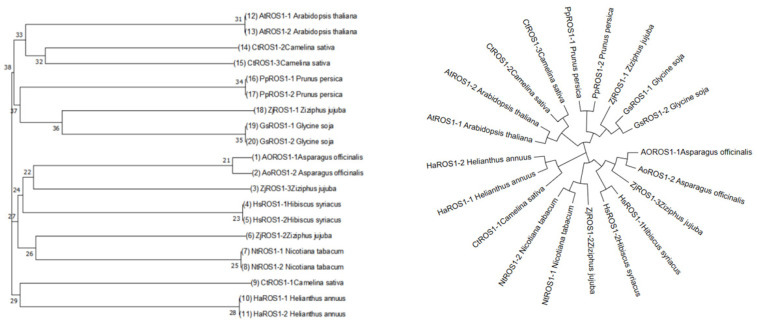
Evolutionary tree of the ROS1 gene family.

The ZjROS1 protein sequence was input into MEGA 7 for a multiple sequence comparison, and a *ZjROS1* gene family evolutionary tree was constructed using the maximum likelihood method. Meanwhile, the ROS1 protein family of *A. thaliana*, *Nicotiana tabacum*, *Glycine soja*, *Asparagus officinalis*, *Prunus salicina*, *Manihot esculenta*, *Camelina sativa*, and *Helianthus annuus* and other ROS1 protein families were introduced, and the evolutionary tree shown in Figure 4 was obtained through an evolutionary analysis. It can be seen in the figure that the three protein sequences of *Z. jujuba* cv. Dongzao are in the same branch as the ROS proteins of *N. tabacum*, *G. soja*, *A. officinalis*, and other plants, and their similarity is high.

#### 3.1.6. Protein Interaction Prediction Analysis

**Figure 5:**
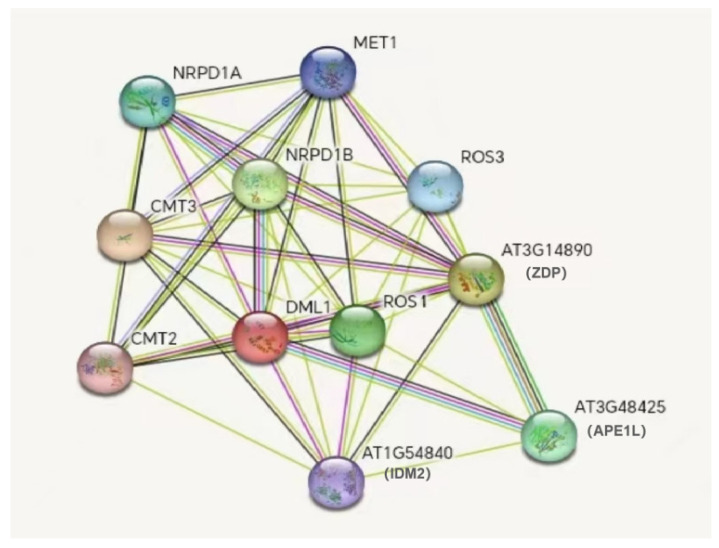
Protein network prediction of ZjROS1 protein function.

The protein sequence was submitted to the STRING online website, the species was selected as the model plant *A. thaliana*, and the prediction results of interactions with the ROS1 protein were obtained (Figure 5). According to the results, with the ROS1 protein as the center, it interacts with the ROS3, CMT2, CMT3, NRPD1B, NRPD1A, MET1, ZDP, IDM2, and APE1L proteins.

### 3.2. Cloning of ZjROS1 Gene of Ziziphus jujuba cv. Dongzao

**Figure 6:**
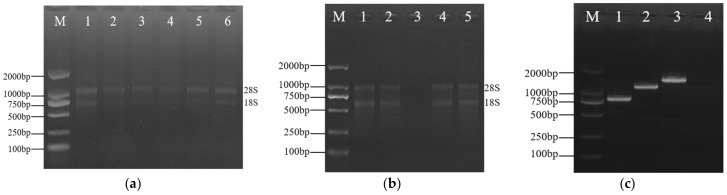
RNA extraction and cloning of the *ZjROS1* gene from *Z. jujuba* cv. Dongzao: (**a**,**b**) RNA extraction from different tissues and fruits; (**a**) lanes 1–6 are for young leaves, old leaves, flowers, white ripe fruits, semi red fruits, and fully red fruits; (**b**) lanes 1–2 and 4–5 are for young stems, old stems, flowers, and white ripe fruits; (**c**) lanes 1–3 are *ZjROS1* gene cloning (lane 1: *ZjROS1-3* 827 bp, lane 2: *ZjROS1-2* 1476 bp, lane 3: *ZjROS1-1* 1656 bp, lane 4: negative control.).

RNA was extracted from different parts and fruits of *Z. jujuba* cv. Dongzao and reverse-transcribed into cDNA. The 28S and 18S RNA bands were clear, and the OD_260_/OD_280_ values were between 1.8 and 2.1, indicating that the extracted RNA had good integrity (Figure 6a,b). Using *Z. jujuba* cv. Dongzao cDNA as a template, PCR was performed on the *ZjROS1* family genes. The obtained bands of the amplified products are shown in Figure 6c.

### 3.3. Analysis of the Tissue Expression Characteristics of ZjROS1 Gene Family

**Figure 7:**
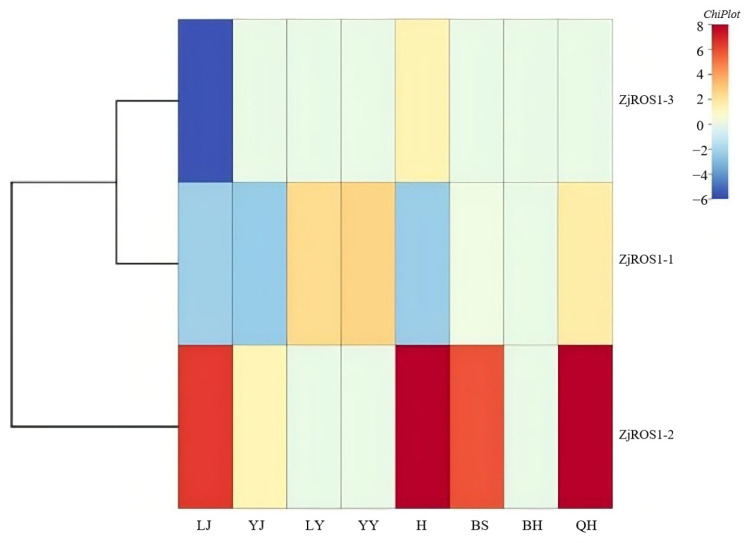
Expression specificity of the *ZjROS1* gene family in *Z. jujuba* cv. Dongzao. LJ: old stem; YJ: young stem; LY: old leaf; YY: young leaf; H: flower; BS: white ripening period; BH: half-red period; QH: full-red period.

The expression of the *ZjROS1* gene family in *Z. jujuba* cv. Dongzao was further studied, the expression and response characteristics of the *ZjROS1* gene in different tissues were analyzed, and a heatmap was drawn (Figure 7). *ZjROS1-1* and *ZjROS1-2* used the semi-red stage as a basis, while *ZjROS1-3* used the young stem as a basis. The results show that the *ZjROS1* gene family was expressed to varying degrees in the stems and flowers, while most genes had low or no expression in the fruits and leaves. In various parts of the *Z. jujuba* cv. Dongzao leaves, except for *ZjROS1-1*, which was expressed in both old and young leaves, the rest were not expressed. In the stems and flowers of *Z. jujuba* cv. Dongzao, the *ZjROS1* gene was expressed to varying degrees, and the expression levels of *ZjROS1-1* and *ZjROS1-2* were higher in the old stems than in the young stems, with *ZjROS1-2* having the highest expression level in the flowers. In the fruits at different stages, except for *ZjROS1-3*, which was not expressed, all other *ZjROS1* genes had a certain expression level, and *ZjROS1-2* had the highest relative expression level during the full-red stage. In addition, the closer the genetic evolutionary relationship, the more similar the expression patterns. The research results indicate that these genes have specific expression patterns in different tissue parts and play a certain role in regulating the maturity and development of *Z. jujuba* cv. Dongzao.

### 3.4. Construction of Ziziphus jujuba cv. Dongzao ZjROS1 Gene-Editing Vector

**Figure 8:**
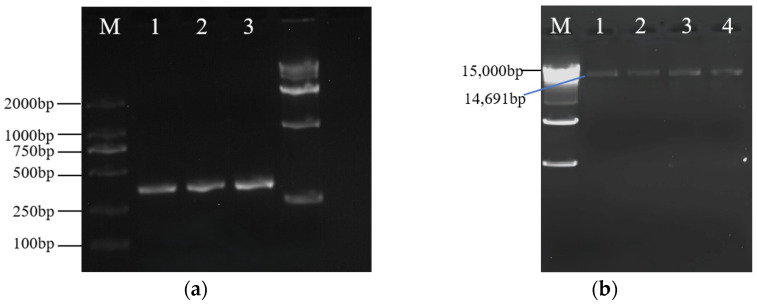
(**a**) SgRNA amplification; (**b**) linearization vector.

The online tool CRISPR2 was used to design gene knockout primers, and the knockout sites were named *ZjROS1-1-1*, *ZjROS1-1-2*, *ZjROS1-2-1*, and *ZjROS1-2-2*. The sgRNA clone frame was obtained via PCR amplification using U6p.4-F universal primers and target gene primers. After amplification, the PCR products were detected using 1% agarose gel electrophoresis, and the target fragment was about 350 bp, which was consistent with the expected results (Figure 8a). A new gene-edited recombinant plasmid was obtained by connecting the pP1C.4 vector cut by *EcoRI* and *XbaI* with the sgRNA frame. The length of the linear vector fragment after digestion was about 14 kb (Figure 8b).

**Figure 9:**
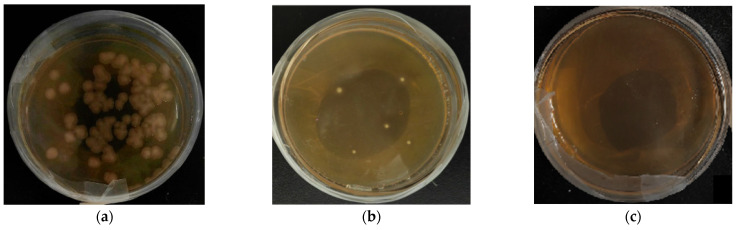
Transformation of *Agrobacterium* GV3101 competent cell colony growth chart. (**a**) Positive control: *Agrobacterium* tumefaciens known to contain the target gene and with a relatively high transformation efficiency was spread on a plate as a positive control. A large number of resistant *Agrobacterium* colonies grew, indicating that the transformation experimental system was basically normal. (**b**) Transforming GV3101 competent cells. (**c**) Negative control. No colonies grew on the negative control plate, indicating that the concentration of the antibiotic used could effectively inhibit the growth of untransformed *Agrobacterium*. This ensures that only *Agrobacterium* that has successfully incorporated the vector containing the resistance gene can grow during the screening of transformants, improving the accuracy of the screening.

**Figure 10:**
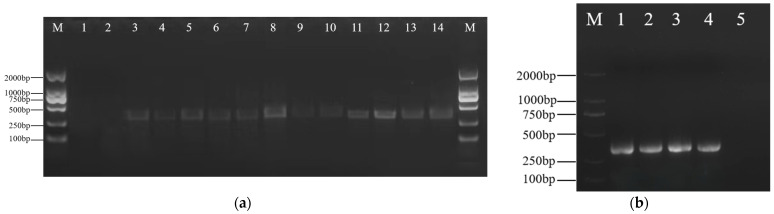
Colony PCR amplification electrophoresis image: (**a**) *Escherichia coli* colony PCR. (lines 1–2: negative control); (**b**) *Agrobacterium* colony PCR (lane 5: negative control). M: DL2000 DNA Marker.

The recombinant gene-editing vector was introduced into *Escherichia coli* GT115 competent cells (Figure 9a–c), single colonies were selected for PCR detection and sequencing identification, and the PCR products were subjected to 1% agar gel electrophoresis. The results show that the target fragment was about 400 bp, which is consistent with the expected results, and it was proven to be a positive clone (Figure 10a). The plasmid of positive *E. coli* bacteria was extracted and introduced into *Agrobacterium* GV3101 receptor cells for PCR verification. The product was subjected to 1% agarose gel electrophoresis, and the results show that the target fragment was about 400 bp, which is consistent with the expected results (Figure 10b), providing a basis for the subsequent genetic transformation.

## 4. Discussion

ROS1 family proteins, also known as DNA demethylases, participate in the demethylation process of DNA methylation modification, which has an important impact on plant growth and development and environmental adaptation [40]. *ROS1* affects gene expression levels by removing 5-methylcytosine from DNA. In addition, the *ROS1* gene also interacts with DNA methylation-modified transcription factors and epigenetic modifiers to regulate gene expression patterns.

Studies have shown that demethylase *ROS1* can regulate various biological processes in many plants and participate in the regulation of plant gene expression [41]. Based on previous studies, in this study, the *AtROS1* gene of *A. thaliana* was compared with the whole genome database of *Z. jujuba* cv. Dongzao; a total of three ZjROS1 proteins with similar domains were compared, and three ZjROS1 proteins were bioinformatically analyzed. The results showed that *ZjROS1* belonged to hydrophilic lipopolysin. Subcellular localization showed that *ZjROS1* was located in the nucleus or cytoskeleton, vacuole, or chloroplast and had no transmembrane domain. It was found that the three proteins were mainly distributed on three chromosomes, and there was no gene tandem replication. By constructing an evolutionary tree, it was found that the three protein sequences were in the same branch as the ROS proteins of *Nicotiana tabacum*, *Glycine soja*, *Asparagus officinalis*, and other plants, and their similarity was high. The RRM_DME complex domain was also found in the conserved structural elements of the ZjROS1 protein sequence, which is involved in DNA demethylation in plants. The *Perm-CXXC* domain catalyzes the release of 5-methylcytosine from DNA through the glycosylase/lyase mechanism. Zhu, M.Y. et al. elaborate in detail on the functions of conserved domains in plant DNA demethylases, including the *RRM_DME* and *Perm-CXXC* domains. Their study showed that the *RRM_DME* domain plays a crucial role in recognizing and binding to specific DNA sequences, which corresponds to the function of the *RRM_DME* complex domain in the ZjROS1 protein in this study, which is involved in DNA demethylation. The *Perm-CXXC* domain has catalytic activity and can participate in the removal process of 5-methylcytosine, similar to the function of the *Perm-CXXC* domain in the ZjROS1 protein in this study, which catalyzes the release of 5-methylcytosine from DNA through the glycosylase/lyase mechanism [42].

By predicting the promoter of the *ZjROS1* gene family in *Z. jujuba* cv. Dongzao, in addition to the promoter necessary for transcription initiation sites, many hormone-related regulatory elements were identified, such as the CGTCA-motif and TGACG-motif of the methyl jasmonate response, suggesting that ZjROS1 may respond to the regulation of methyl jasmonate. It was involved in the endosperm expression element, that is, the GCN4_motif, and the meristemia-specific element, that is, the CAT-box, indicating that the *ZjROS1* gene family may be involved in plant cell growth and fruit development. The elements associated with biotic and abiotic stresses indicate that the *ZjROS1* gene family may respond to drought stress, low temperature, etc. Zhu, H.F. et al. found that ROS1 negatively regulates the DNA methylation of the *DOGL4* promoter, thereby controlling gene imprinting. Moreover, it regulates seed dormancy and the *ABA* response through *DOGL4*. In the plant hormone regulation network, there may be certain interactions between *ABA* and methyl jasmonate, which indirectly indicates the role of the *ROS1* gene in related regulatory pathways. This study also pointed out that *DOGL4* is an imprinted gene in the endosperm of *A. thaliana*. *ROS1* negatively regulates *DOGL4* gene imprinting by preventing the hypermethylation and complete silencing of the paternal allele, affecting its expression in the endosperm. Additionally, this study discovered that *ROS1* affects seed dormancy by regulating *DOGL4* gene imprinting. As seed dormancy is closely related to plant growth and development, it indirectly reflects the influence of the *ROS1* gene on plant growth and development, which corresponds to the promoter prediction results of this study [43]. Chang, Y.N. et al. found that *ROS1*-mediated DNA demethylation is involved in the regulation of the stress response, further demonstrating the epigenetic regulatory role of the *ROS1* gene in the plant stress response [44]. In conclusion, combined with the promoter element analysis, it is indicated that the *ZjROS1* family may participate in the development and ripening of *Z. jujuba* cv. Dongzao fruits through multiple pathways.

A protein interaction network prediction analysis revealed that the ROS1 protein interacts with the DML1, CMT2, and CMT3 proteins. The tissue expression characteristics of the *ZjROS1* gene family were analyzed, and the results show that the *ZjROS1* gene family is specifically expressed in different tissue of *Z. jujuba* cv. Dongzao, which is speculated to play a certain role in the regulation of the maturation and development of *Z. jujuba* cv. Dongzao.

In plants, active DNA demethylation is initiated by the *ROS1* family with bifunctional DNA glycosylase/lyase. *ROS1* has been shown to play an important regulatory role in the demethylation mechanism of plant DNA [45]. In *Nicotiana tabacum*, *NtROS1* has also shown the ability to remove 5-methylcytosine from DNA in addition to 5-methylcytosine, but this reaction is less efficient than 5-methylcytosine because the plant rarely uses 5-methylcytosine as an epigenetic mark [46]. In *Oryza sativa*, it was found through gene cloning that a dominant negative mutation of the DNA demethylase gene *OsROS1* led to aleurone layer thickening, which is of great significance for expanding the breeding of the nutritional quality of gramineous crops [47]. At present, there are few studies on the effects of *ROS1* on plant growth and development. Using CRISPR/Cas9 gene-editing technology to select alleles derived from DNA methylation or demethylation mutants or to generate alleles as editing sites to obtain mutant plants is an important method for plant breeding [41].

In the future, we plan to use CRISPR/Cas9 technology to obtain gene-edited plants and further study the specific functions of the *ZjROS1* gene family members in the growth and development, fruit quality formation, and stress resistance of *Z. jujuba* cv. Dongzao. By comparing the phenotypic differences between wild-type and gene-edited plants at different growth stages and under different environmental conditions, as well as combining the determination of physiological and biochemical indicators, the functional mechanism of the *ZjROS1* gene will be clarified. We will search for the upstream transcription factors that regulate the expression of the *ZjROS1* gene, as well as the downstream target genes regulated by the *ZjROS1* gene; draw a complete regulatory network; and deeply examine its action path in the biological processes of *Z. jujuba* cv. Dongzao. By combining multi-omics technologies, such as transcriptomics, proteomics, and metabolomics, we will comprehensively analyze the role of the *ZjROS1* gene family in the growth, development, and environmental response of *Z. jujuba* cv. Dongzao. Through an integrated analysis of multi-omics data, we will explore the key metabolic pathways and biological processes related to the *ZjROS1* gene, discover more potential regulatory factors and functional genes, attempt to genetically modify *Z. jujuba* cv. Dongzao through gene-editing technology, cultivate new varieties with more excellent traits, and promote the development of the *Z. jujuba* cv. Dongzao industry.

## 5. Conclusions

DNA demethylase essentially contains *ENDO3C*, *RRM-DME*, and *Perm-CXXC* domains, which are closely related to plant growth and development regulation, plant stress resistance, etc. In this study, we identified three *ZjROS1* genes with the *RRM-DME* and *Perm-CXXC* domains. A gene-editing vector was constructed, laying the foundation for subsequent genetic transformation. A tissue expression specificity analysis revealed that the *ZjROS1* gene was related to the growth and development of *Z. jujuba* cv. Dongzao fruit. *ZjROS1* may regulate the growth and development of *Z. jujuba* cv. Dongzao fruit through DNA methylation modification, but the specific methylation function and epigenetic regulatory mechanism of *Z. jujuba* cv. Dongzao need to be further verified. By constructing a phylogenetic tree, it was found that compared with the *ROS1* genes in other species, the *ZjROS1* gene in *Z. jujuba* cv. Dongzao exhibits a certain evolutionary conservation. This implies that during the long evolutionary process, the *ROS1* genes of these plants may have originated from a common ancestor, and there may also be similarities in their functions. For instance, in *A. thaliana*, the *ROS1* gene plays a crucial regulatory role in coping with abiotic stress. It promotes the expression of defense genes and stress-responsive genes by reducing the DNA methylation level. Although the *ZjROS1* gene in *Z. jujuba* cv. Dongzao shares similarities with the *ROS1* genes of these species, due to the unique growth characteristics and fruit development process of *Z. jujuba* cv. Dongzao, the *ZjROS1* gene may possess unique functions in regulating the fruit ripening and development of *Z. jujuba* cv. Dongzao. In subsequent research, based on the constructed gene-editing vector, the CRISPR/Cas9 technology can be utilized to obtain gene-edited plants. By comparing the differences in aspects such as growth and development, fruit quality, and stress resistance between wild-type and gene-edited plants, the specific functions of the *ZjROS1* gene family members can be explored in depth.

This study provides an important theoretical basis for further understanding the regulatory mechanism of *ROS1* demethylation and its roles in the growth and development of *Z. jujuba* cv. Dongzao.

## Figures and Tables

**Table 1:** Primer sequences used in the experiment.

Primer Name	Primer Sequence (5′–3′)	Primer Use
ZjROS1-1-F	ATGTCTTTCACTGGCCTATTGG	Gene clone
ZjROS1-1-R	CTACTCATCTTTCCTTTCCTTATTT	
ZjROS1-2-F	ATGTAGTGGAACTAATCATGTTTGC	
ZjROS1-2-R	CTATCTCCTTGAACAAGATGCATT	
ZjROS1-3-F	ATGCTGAACCCATCATTGAAGT	
ZjROS1-3-R	CTACTCATCATCGTCTTCGTTTATC	
dl-ROS1-1-F	TGCACCTGTAACACCGGATA	Real-time fluorescence quantitative PCR reaction
dl-ROS1-1-R	GGAATCTGAAGCAGGCTTTG	
dl-ROS1-2-F	GAACCAAACGGGTAAAGCAA	
dl-ROS1-2-R	TTGTGCTGCCATTTTGAGAG	
dl-ROS1-3-F	AGGCAAGTTCAAAAGCTCCA	
dl-ROS1-3-R	CTCACAAGATGCTGGCTCTG	
ZjACT-F	TCACACTTTCTACAATGAGCT	
ZjACT-R	ATATCCACATCACACTTCAT	

**Table 2:** Primer sequences.

Primer Name	Primer Sequence (5′–3′)	Primer Use
qc-ROS1-1-1 R	GCTATTTCTAGCTCTAAAACTGGTCACCTCTTTCAGCTACAATCACTACTTCGACTCT	Gene editing
qc-ROS1-1-2 R	GCTATTTCTAGCTCTAAAACAGTCTGGAAGTTCATAGACTCAATCACTACTTCGACTCT	
qc-ROS1-2-1 R	GCTATTTCTAGCTCTAAAACCTTTCTTCACTAGACTTGGGCAATCACTACTTCGACTCT	
qc-ROS1-2-2 R	GCTATTTCTAGCTCTAAAACTTTGGATTGACATCATTTGCAATCACTACTTCGACTCT	
U6p.4-F	CAGGAAACAGCTATGACCATATTCATTCGGAGTTTTTGTATC	

**Table 3:** Physicochemical properties of ZjROS1 protein in *Z. jujuba* cv. Dongzao.

Gene NAME	Sequence ID (NCBI)	Number of Amino Acids (aa)	Molecular Weight (Da)	Theoretical pI	Instability Index	Aliphatic Index	Grand Average of Hydropathicity
*ZjROS1-1*	XP_015898699.1	1758	197,208.61	6.49	46.90	65.63	−0.768
*ZjROS1-2*	XP_015876562.1	1938	217,902.56	7.69	47.56	68.98	−0.767
*ZjROS1-3*	XP_024926338.1	753	84,959.77	5.74	54.16	73.84	−0.584

**Table 4:** Prediction of the protein secondary structure of the *ZjROS1* family.

Gene	α Helix	β Turn	Extended	Random Coil
*ZjROS1-1*	32.31%	6.68%	15.47%	45.34%
*ZjROS1-2*	34.31%	6.66%	14.04%	44.99%
*ZjROS1-3*	35.99%	7.97%	14.08%	41.97%

## Data Availability

The datasets supporting the results presented in this manuscript are included within the article (and its Supplementary Materials).

## Supplementary Materials

The following supporting information can be downloaded at: https://www.mdpi.com/article/10.3390/genes16020228/s1. Supplementary Table S1: PCR amplification reaction system and program; Supplementary Table S2: Real-time PCR reaction system and procedure; Supplementary Table S3: High-Fidelity DNA Polymerase PCR amplification reaction system and program; Supplementary Table S4: Enzyme digestion reaction system; Supplementary Table S5: Recombinant enzyme integration reaction system; Supplementary Table S6: Subcellular localization prediction of *ZjROS1* family; Supplementary Figure S1: Prediction of hydrophilicity of ZjROS1 family proteins in *Ziziphus jujuba* cv. Dongzao; Supplementary Figure S2: Prediction of phosphate sites in the ZjROS1 family protein of *Ziziphus jujuba* cv. Dongzao; Supplementary Figure S3: Analysis of ZjROS1 family protein signal peptides in *Ziziphus jujuba* cv. Dongzao; Supplementary Figure S4: Analysis of transmembrane domains of ZjROS1 family proteins in *Ziziphus jujuba* cv. Dongzao; Supplementary Figure S5: Prediction of secondary structures of *ZjROS1-1*, *ZjROS1-2*, and *ZjROS1-3*; Supplementary Figure S6: Prediction of tertiary structures of *ZjROS1-1*, *ZjROS1-2*, and *ZjROS1-3*; Supplementary Figure S7: Plasmid map of pP1C.4.
