# Supplementary material for: Identification of Ziziphus jujuba cv. Dongzao DNA Demethylase ZjROS1 Gene Family and Construction of CRISPR/Cas9-Mediated Gene-Editing Vector
Source: Genes (Basel). 2025 Feb 17;16(2):228. doi: 10.3390/genes16020228 (PMC11855291; doi:10.3390/genes16020228)
Supplement: Supplementary file 1 [file genes-16-00228-s001.zip › Supplementary documents for manuscript 3441288.pdf]

Supplementary Table S1. PCR amplification reaction system and program.

Reagent name	Volume/μL	Reaction program	
2×San Taq PCR Mix	25.0	94℃ 5min	1×
DNA template	4.0	94℃ 30sec	
PCR Forward Primer	2.0	58℃ 30sec	35×
PCR Reverse Primer	2.0	72℃ 1min	
Sterilized ddH_2O	Up to 50.0	72℃ 10min	1×

Supplementary Table S2. Real-time PCR reaction system and procedure.

Reagent name	Volume/μL	Reaction program	
TB Green Premix Ex Taq II (Tli RNaseH Plus)(2×)	10.0		
DNA template	2.0	95℃ 30sec	1×
PCR Forward Primer（10 μM）	0.4	95℃ 5sec	
PCR Reverse Primer（10 μM）	0.4	60℃ 20sec	40×
Sterilized ddH_2O	Up to 20.0		

Supplementary Table S3. High-Fidelity DNA Polymerase PCR amplification reaction system and program.

Reagent name	Volume/μL	Reaction program	
pP1C.4	0.8	94℃ 5min	1×
U6p.4-F Universal primers	0.5	94℃ 30sec	
Oligo-F	0.5	58℃ 30sec	35×
2×TransStart KD Plus PCR SuperMix	5.0	68℃ 1min	
ddH_2O	Up to 10	68℃ 10min	1×

Supplementary Table S4. Enzyme digestion reaction system.

Reagent name	Volume/μL	Reaction conditions
pP1C.4 carrier	5.0	37℃ 1h Incubation
1×Buffer Y	2.0	
EcoRI	1.0	
XbaI	1.0	
ddH_2O	Up to 20	

Supplementary Table S5. Recombinant enzyme integration reaction system.

Reagent name	Volume	Reaction conditions
G-force Buffer solution (5×)	2.0μL	37℃ 30 min Incubation<br>Quickly place on ice 15 min
G-force Recombinant enzyme	1.0μL	
Linearized pP1C carrier	100 ng	
Amplified fragment product	5~10 ng	
ddH_2O	Up to 10μL	

Supplementary Table S6. Subcellular localization prediction of *ZjROS1* family.

Gene Name	Subcellular localization
ZjROS1-1	nucl: 14
ZjROS1-2	nucl: 13, cysk: 1
ZjROS1-3	nucl: 7, cysk: 1, vacu: 1, chlo: 5

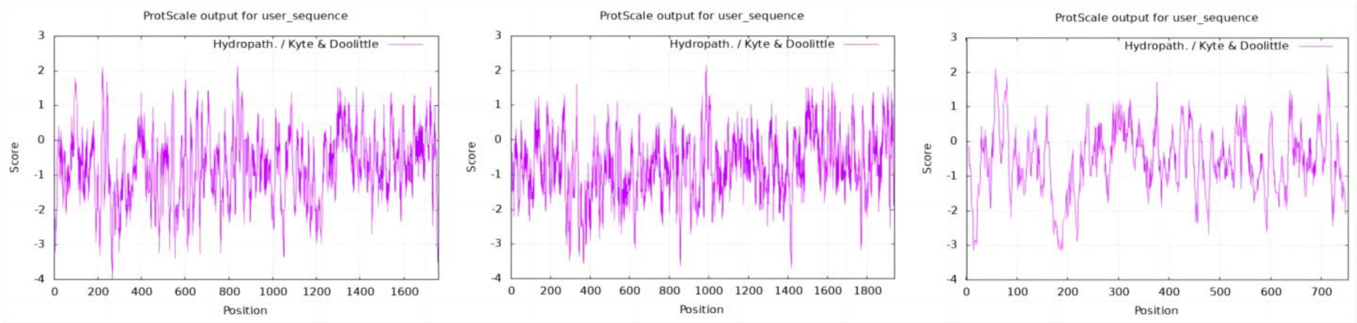

Supplementary Figure S1. Prediction of hydrophilicity of ZjROS1 family proteins in *Ziziphus jujuba* cv. Dongzao.

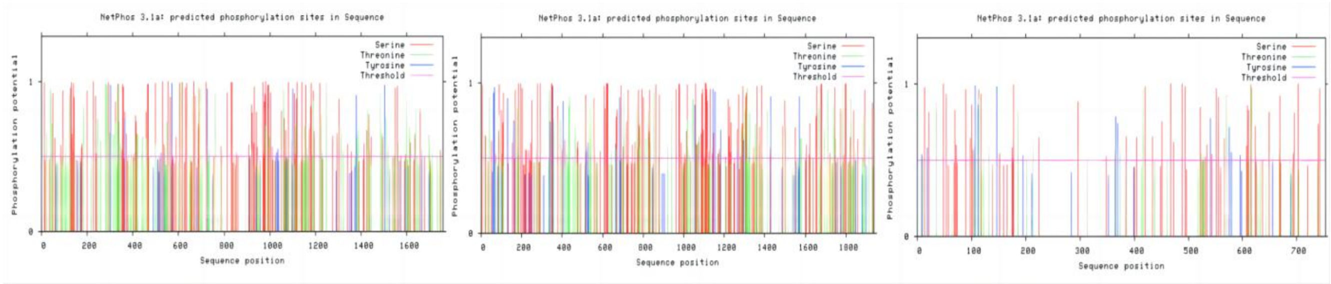

Supplementary Figure S2. Prediction of phosphate sites in the ZjROS1 family protein of *Ziziphus jujuba* cv. Dongzao.

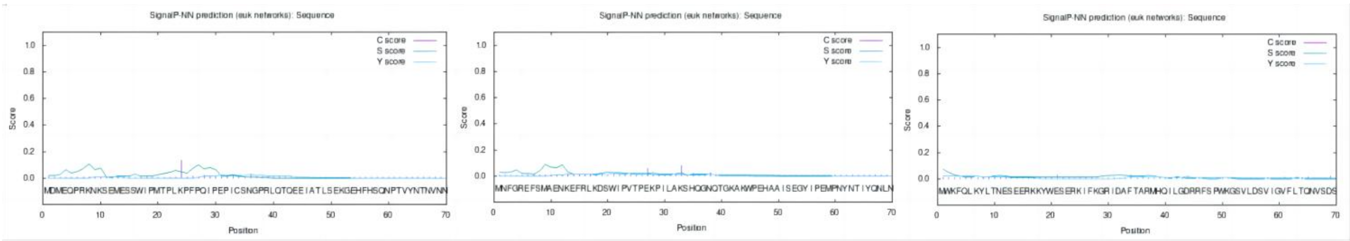

Supplementary Figure S3. Analysis of ZjROS1 family protein signal peptides in *Ziziphus jujuba* cv. Dongzao.

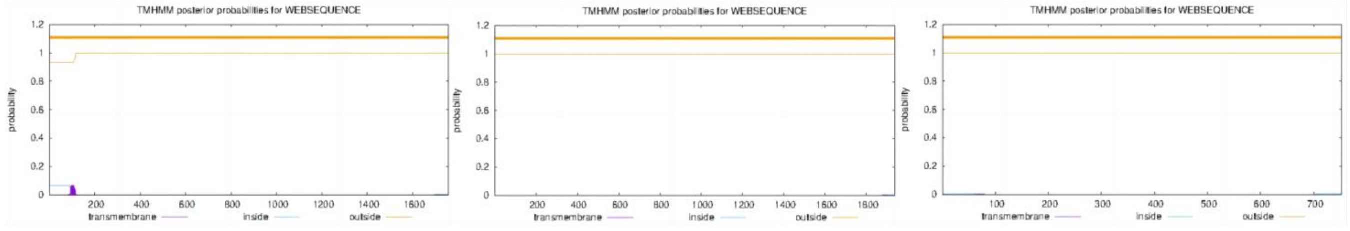

Supplementary Figure S4. Analysis of transmembrane domains of ZjROS1 family proteins in *Ziziphus jujuba* cv. Dongzao.

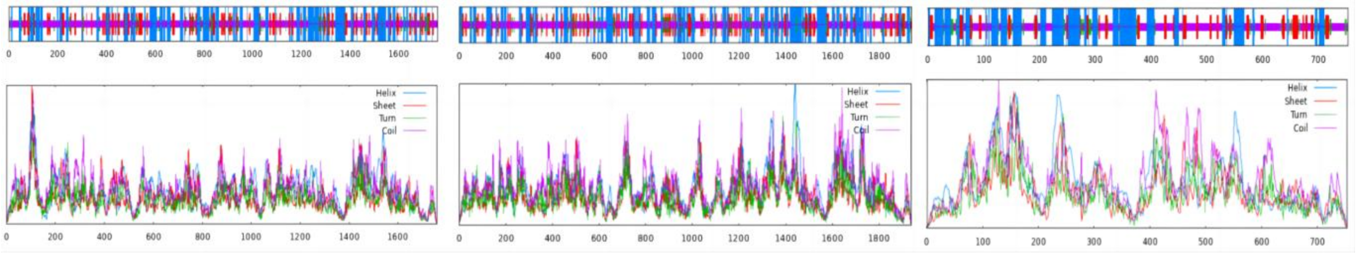

Supplementary Figure S5. Prediction of secondary structures of *ZjROS1-1*、*ZjROS1-2*、*ZjROS1-3.* The blue color in the figure refers to α- Spiral, red refers to extended chain, green refers to β- Corner, purple refers to irregular curls.

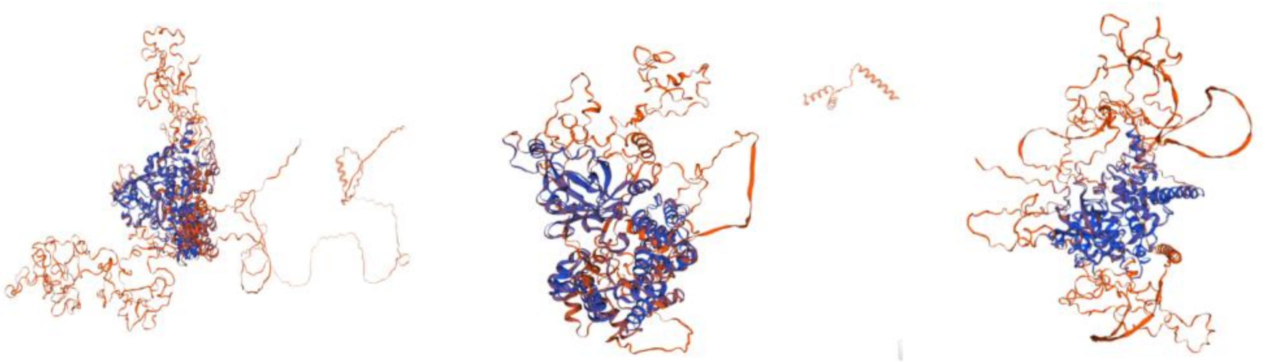

Supplementary Figure S6. Prediction of tertiary structures of *ZjROS1-1*、*ZjROS1-2*、*ZjROS1-3.*

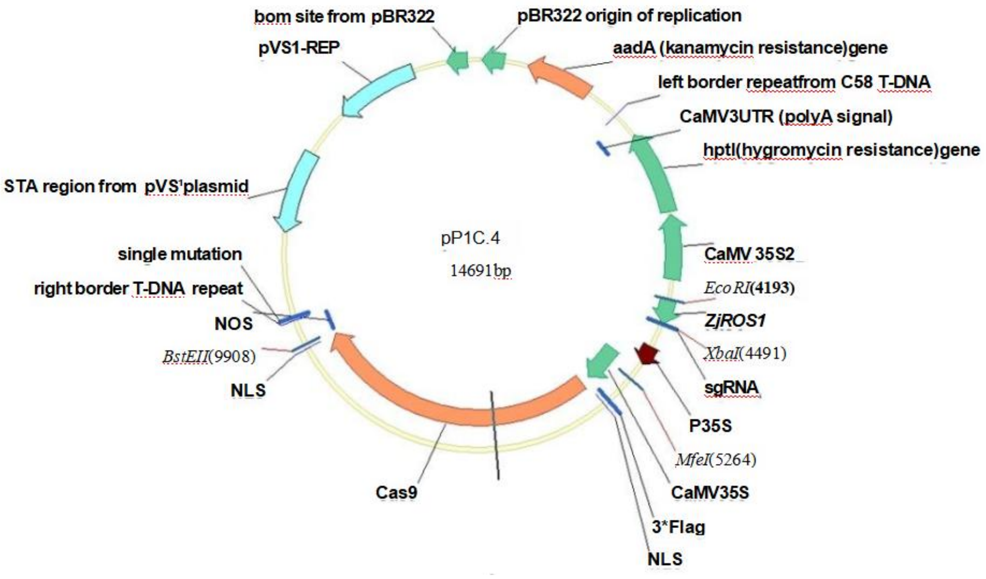

Supplementary Figure S7. Plasmid map of pP1C.4.
